# Novel machine learning fusion architectures integrating electrocardiogram representations: applications to acute coronary event detection

**DOI:** 10.1093/ehjdh/ztag062

**Published:** 2026-05-08

**Authors:** Rui Qi Ji, Nathan T Riek, Tanmay Gokhale, Zeineb Bouzid, Karina Kraevsky, Jessica K Zègre-Hemsey, Samir Saba, Christian Martin-Gill, Clifton W Callaway, Murat Akcakaya, Ervin Sejdić, Salah S Al-Zaiti

**Affiliations:** The Edward S. Rogers Sr, Department of Electrical & Computer Engineering, University of Toronto, Toronto M5S 1A1, Ontario, Canada; School of Nursing, University of Rochester, Rochester, NY 14627, USA; University of Pittsburgh Medical Centre, Pittsburgh, PA 15260, USA; School of Electrical and Computer Engineering, Georgia Institute of Technology, Atlanta, GA 30332, USA; University of Pittsburgh Medical Centre, Pittsburgh, PA 15260, USA; School of Nursing, University of North Carolina at Chapel Hill, Chapel Hill, NC 27599, USA; University of Pittsburgh Medical Centre, Pittsburgh, PA 15260, USA; University of Pittsburgh Medical Centre, Pittsburgh, PA 15260, USA; University of Pittsburgh Medical Centre, Pittsburgh, PA 15260, USA; Department of Electrical and Computer Engineering, University of Pittsburgh, PA 15260, USA; The Edward S. Rogers Sr, Department of Electrical & Computer Engineering, University of Toronto, Toronto M5S 1A1, Ontario, Canada; North York General Hospital, Toronto M2K 1E1, Canada; School of Nursing, University of Rochester, Rochester, NY 14627, USA

**Keywords:** Electrocardiogram, Machine learning, Fusion learning, Acute coronary events, Diagnosis, Emergency department triage

## Abstract

**Aims:**

AI in electrocardiography (ECG) has diverged into two paths: traditional signal processing with machine learning, and deep learning of raw waveforms. The former preserves physiological interpretability but may miss novel patterns, while the latter overlooks a century of ECG science. No framework yet integrates both. This study introduces a fusion model combining handcrafted feature-based random forest and waveform-based convolutional neural network to improve the diagnosis of acute coronary events.

**Methods and results:**

This prospective, multicentre cohort study included pre-hospital ECGs recorded on emergency medical services from patients with a chief complaint of chest pain. Using this dataset, we developed four fusion frameworks that integrate ECG features with waveform information: (1) decision-level fusion combining predictions from random forest and convolutional neural network (CNN); (2) decision-level fusion with retraining; (3) feature-level fusion by concatenating CNN embeddings with ECG features; and (4) feature-level fusion combining CNN embeddings with tree-level outputs from the random forest. This study comprised of 10 393 ECGs from 7397 patients. On both outcomes, acute coronary syndrome and occlusion myocardial infarction, the proposed decision fusion with retraining (Approach 2) achieved an AUROC of 0.878 (95% CI, 0.837–0.915) and 0.961 (95% CI, 0.934–0.983) on the test set and an average precision score of 0.749 (95% CI, 0.681–0.810) and 0.850 (95% CI, 0.763–0.918), respectively, outperforming all other proposed fusion and individual models.

**Conclusion:**

This study demonstrates the significance of fusion models that integrate complementary ECG representations to improve diagnostic accuracy in acute coronary events, highlighting a promising approach for real-time AI-augmented cardiovascular disease detection across diverse use cases.

## Graphical Abstract

**Graphical Abstract:**
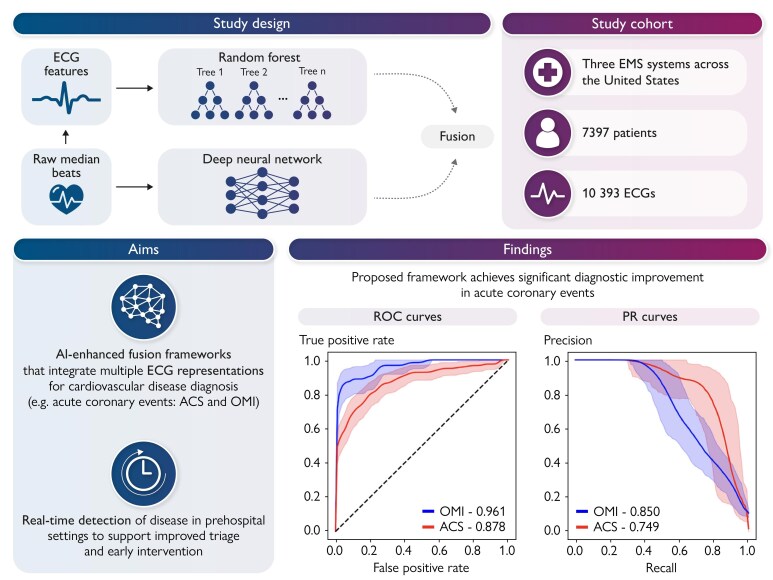

## Introduction

Artificial intelligence (AI) applications in electrocardiography (ECG) have rapidly advanced and revolutionized real-time clinical decision-making,^1,2^ yet current approaches remain polarized between two extremes: Traditional signal processing–based feature extraction coupled with machine learning, and end-to-end deep learning applied directly to raw waveforms.^3^ The former builds upon nearly a century of physiological insight and proven interpretability but may overlook subtle, previously unrecognized waveform signatures. The latter, while powerful, often dismisses decades of foundational ECG research and risks reducing physiological understanding to a data-driven black box. Despite their complementary strengths, there are currently no established frameworks to effectively harness both paradigms.^4–6^ Fusion learning provides an opportunity to bridge this divide by integrating interpretable, handcrafted features with high-dimensional deep learning representations.

In our previous work, we demonstrated that AI-enhanced ECG can accurately predict acute coronary events, including acute coronary syndrome (ACS) and occlusion myocardial infarction (OMI), but these studies used either a feature-based random forest model or a waveform-based convolutional neural network (CNN) model.^7–9^ Our random forest model trained on handcrafted ECG features achieved strong performance for ACS and OMI prediction, and our CNN model, ECG-SMART-NET, trained on median beats (i.e. single representative waveforms derived from a 10-s ECG), outperformed prior approaches in OMI detection by learning directly from raw signals. These findings highlight the complementary strengths of each approach: random forests excel with engineered, clinically informed features, while CNNs automatically extract patterns from raw signals. However, combining deep learning models with non-differentiable machine learning models, such as random forests, poses unique challenges, as integrated gradient-based training is not possible.^10^ This motivates the exploration of fusion approaches that integrate different models and ECG representations to enhance diagnostic accuracy.^11^

ACS and OMI are particularly suitable use cases for fusion learning. ACS occurs when coronary artery obstruction restricts blood flow to the heart, leading to myocardial injury,^12,13^ and OMI, a complete blockage, is a time-critical subset requiring immediate intervention.^14^ These conditions exemplify structural and functional changes in the heart, which are reflected in both subtle waveform patterns across leads and derived features such as QRS duration, ST-segment deviations, or T-wave morphology.^15,16^ Thus, ECG screening is often the first step in evaluating patients with chest pain, but traditional interpretation focusing on ST-segment elevation MI (STEMI) misses many OMI cases.^17–19^ Subtle or absent classical ECG signs can delay diagnosis and treatment, and accurate early differentiation between OMI and non-OMI ACS is essential, as management strategies differ.^20–22^ Fusion learning is thereby a promising solution to the diagnosis of such conditions: by learning from raw waveforms, deep learning models can capture complex, high-dimensional temporal and spatial relationships, while feature-based models leverage established clinical markers that are interpretable and robust. Fusion of these complementary approaches can provide more precise and reliable detection of ACS and OMI, offering the potential to improve early diagnosis, risk stratification, and real-time triage in pre-hospital and hospital settings.

In this study, we introduce novel fusion architectures that combine a feature-based random forest with a waveform-based CNN to enhance diagnostic accuracy in acute coronary events, specifically ACS and OMI, where rapid and precise diagnosis is paramount. To our knowledge, this is the first study that proposes machine learning and deep learning fusion approaches in ECG and cardiovascular diagnostic applications. We systematically compare their performance in ACS and OMI diagnostic applications to assess not only whether fusion improves accuracy, but also how different fusion mechanisms influence learning dynamics and diagnostic value. While ACS and OMI serve as the primary use cases in this study, these fusion frameworks are broadly applicable to the detection of other cardiovascular diseases, as the diagnoses of many conditions can benefit from integrating both waveform patterns and quantitative ECG features.

## Methodology

### Study population and outcome adjudication

The dataset used in this study consists of pre-hospital ECGs for patients with a primary complaint of acute chest pain transported by emergency medical services (EMS) in three US cities: Pittsburgh, PA, Charlotte, NC, and Chapel Hill, NC.^8,23–25^ The ECGs were recorded using Heart Start MRX (Philips Healthcare) devices in Pittsburgh and Charlotte and using LIFEPAK 15 (Stryker) devices in Chapel Hill. In total, 11 309 ECGs were collected.

We removed 93 ECGs with ventricular tachycardia and 115 ECGs that had more than one missing or noisy ECG lead (or if lead I, II, V1, or V6 was missing/noisy). Additionally, serial ECGs were retained in training to increase sample diversity, but were removed from the validation and test sets to prevent inflating performance metrics. A total of 10 393 ECGs were used in this study. Next, patients were split into 80% training, 10% validation, and 10% test sets. The splits were stratified so that the disease prevalences across the splits were representative of the overall prevalence. This split was retained from our previous work to maintain consistency and comparability.^9^  *Table 1* provides more details about the number of patients and disease prevalence in each split.

**Table 1:** OMI and ACS prevalence of each split

Split	ECGs	Patients	ACS [*n* (%)]	OMI [*n* (%)]
Training	8912	5916	901 (15.2)	411 (6.9)
Validation	740	740	121 (16.4)	57 (7.7)
Test	741	741	125 (16.9)	62 (8.4)
Total	10 393	7397	1147 (15.5)	530 (7.2)

This study complies with all ethical regulations and was approved by the institutional review board of the University of Pittsburgh. The study was registered on ClinicalTrials.gov [ID: NCT04237688].

### Outcome definition

The outcomes of interest were ACS and OMI. ACS (i.e. evidence of myocardial infarction or unstable angina) was defined by having ischaemic symptoms (e.g. chest pain, breathlessness, etc.) and meeting at least one of the three criteria: (1) elevated cardiac troponin I (>99th percentile), (2) imaging evidence of new myocardial injury, or (3) > 70% coronary narrowing. OMI was defined as any patient with a Thrombolysis In Myocardial Infarction (TIMI) flow grade of 0–1 or any patient with a TIMI flow grade of 2 with coronary narrowing >70% and a peak cardiac troponin I of at least 5–10 ng/mL.^26^ By definition, all patients with OMI also have ACS, but not all patients with ACS have OMI. Thus, our goal is to develop a framework that can be trained and evaluated on both outcomes and achieve high diagnostic performance. *Table 2* provides additional information about the included ECGs.

**Table 2:** Summary of baseline characteristics of study cohort

	Study cohort (*n* = 7397)
**Age**	59 ± 16
**Sex**	
Female	3392 (45.9%)
Male	4005 (54.1%)
**Race**	
White	3064 (41.4%)
Black	2894 (39.1%)
Others	93 (1.3%)
Unknown	1346 (18.2%)
**Past medical history**	
Hypertension	2870 (38.8%)
Diabetes	1401 (18.9%)
Current smoker	1181 (16.0%)
High cholesterol	1882 (25.4%)
Known CAD	1375 (18.6%)
Prior MI	954 (12.9%)
Prior PCI	143 (1.9%)
Prior CABG	579 (7.8%)
**ECG and lab findings**	
Sinus rhythm	6029 (81.5%)
Atrial fibrillation	510 (6.9%)
Left BBB	166 (2.2%)
Right BBB	351 (4.7%)
ECG-LVH	661 (8.9%)
**Medical therapy**	
PCI	689 (9.3%)
Total LAD occlusion	330 (4.5%)
Total LCX occlusion	247 (3.3%)
Total RCA occlusion	314 (4.2%)
**Study outcomes**	
ACS	1147 (15.5%)
OMI	530 (7.2%)

### Baseline standalone models

In our previous works,^8,9^ we were able to achieve high performance for OMI prediction using a CNN with median-beat input and a feature-based random forest using 417 ECG features identified by our proprietary pre-processing pipeline. In short, a median beat is a single representative waveform derived from a standard 10-s ECG, designed to reduce noise and highlight the underlying morphology. This provides clearer structural information, which is particularly valuable for detecting conditions such as myocardial infarction.

We performed a comprehensive grid search for CNN and the random forest from prior works for both ACS and OMI outcomes.^8,9^  *Table 1* in the Supplementary section shows the hyperparameters explored in the grid search as well as the optimal hyperparameters for the CNN and the random forest. These hyperparameters were then locked, and the hybrid models proposed in this work were built upon them.

### Rationale for fusion

**Figure 1:**
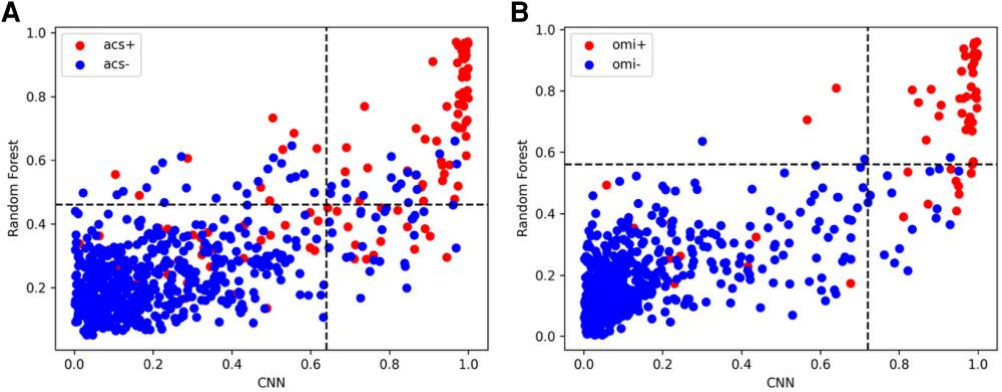
Scatterplots showing decision boundaries for the baseline random forest and baseline CNN in (*A*) ACS and (*B*) OMI outcomes. Each point represents one sample from the test set, with values indicating the predicted likelihood of a positive. Points located to the right or above the decision boundaries were classified as positive cases by the corresponding model, while the colour of each point reflects the true outcome.

The rationale for fusion stems from the observation that our baseline random forest and CNN models perform better on different subsets of cases. Therefore, the goal of the fusion is to leverage the strengths of each model. To demonstrate this, we generated plots illustrating the predictions for the random forest vs. predictions for the CNN for both ACS and OMI on the test set (*Figure 1*). The figure highlights the decision thresholds that separate negative and positive classes for each model, with thresholds chosen to maximize the *F*1 score on the validation set. The decision thresholds for ACS outcome were 0.46 and 0.64 for the random forest and the CNN, respectively. The decision thresholds for OMI outcome were 0.56 and 0.72 for the random forest and the CNN, respectively. As shown in *Figure 1*, each model misclassifies a different subset of cases, underscoring the value of fusion models that can leverage the complementary strengths of both approaches to reduce misclassification rate.

**Figure 2:**
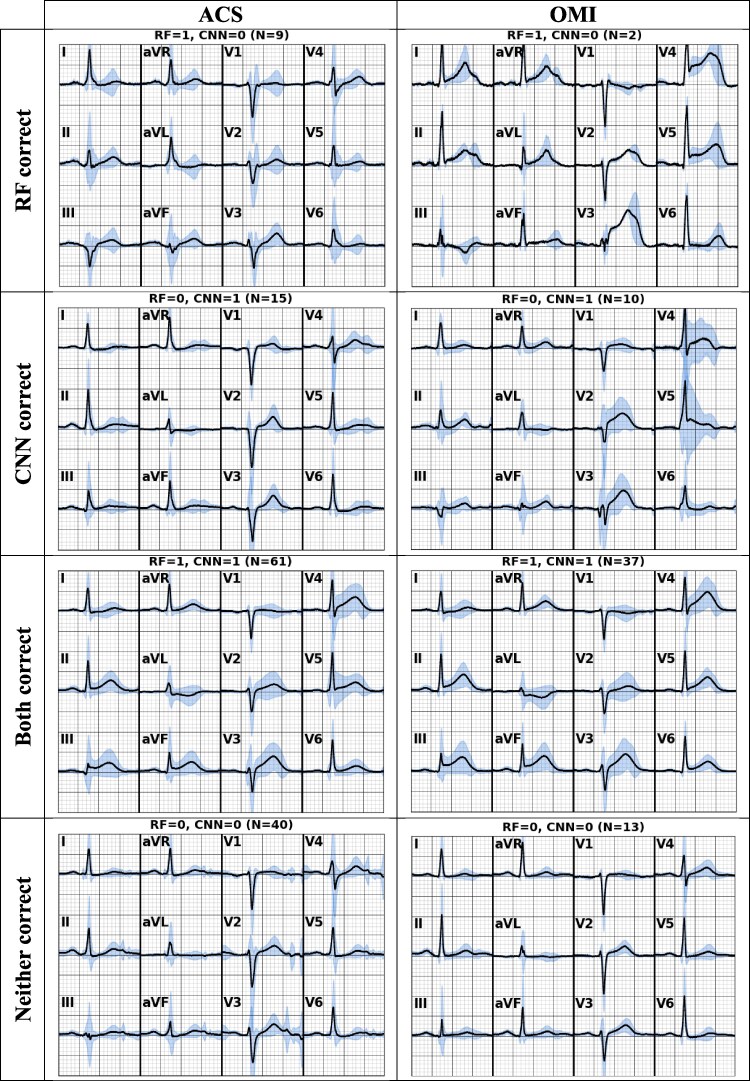
Comparison of averaged ECGs for positive test cases predicted correctly across models. Each column represents a specific outcome, while each row corresponds to whether one model, both models, or neither correctly classified the ECG samples.

To better understand the cases that each model predicted correctly and incorrectly, we also show representative ECGs averaged across true positive cases of ACS (left) and OMI (right), stratified by whether they were correctly classified by the random forest or the CNN model in *Figure 2*. Each panel displays the 12-lead ECG with the mean waveform (black) and confidence intervals (blue shading) across the corresponding subgroup of patients (number of cases indicated above each plot). It is again evident that each of the models misclassified a different subset of cases.

In the case of ACS, the patients with true ACS and discordant predictions seem to have relatively subtle ECG changes (*Figure 2*, left column). Those identified by the random forest model as positive but by the CNN as negative seem to have more T wave flattening/inversions, while those identified by the random forest as negative but by the CNN as positive appear to have more hyperacute T wave changes. In the case of OMI (*Figure 2*, right column), relatively few patients were identified by the random forest model as positive and the CNN as negative, but these patients tended to have dramatic ST changes that may not have been adequately represented in the training set.

### Hybrid models and fusion strategies

**Figure 3:**
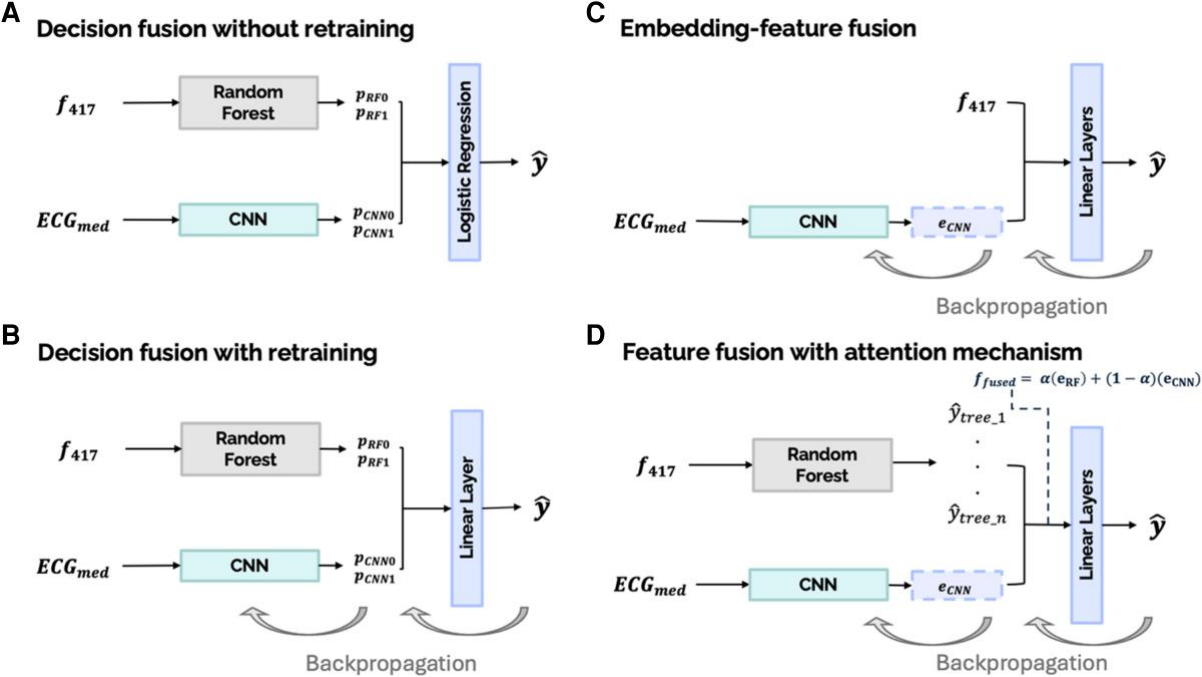
Proposed hybrid model architectures: (*A*) decision fusion without retraining, (*B*) decision fusion with retraining, (*C*) embedding-feature fusion, and (*D*) feature fusion with attention mechanism. ECG_med_ denotes ECG median beats, *f*_417_ denotes the 417 handcrafted features extracted, *p*_0_ denotes probability for the negative class, *p*_1_ denotes probability for the positive class, e_RF_ denotes feature space of the random forest model (probabilistic predictions from each tree), e_CNN_ denotes feature space of the CNN model, *α* denotes the learned attention weight for combining the feature spaces, and *f*_fused_ denotes the combined feature space.

Machine learning models each have their own strengths and weaknesses. Fusion models can leverage the strengths of different models to achieve higher performance than the underlying models individually. Models can be fused either at the decision level or at the feature level.^27^ Decision fusion involves taking the predictions of multiple models and combining these predictions as input to another model. Feature fusion involves taking features from different sources and feeding them into a single model. In this work, we designed both decision-level and feature-level fusion models incorporating the CNN and the random forest from our previous works. The fusion strategies are illustrated in *Figure 3*. To ensure consistency and comparability with individual models, the same hyperparameters were applied in the fusion model.

The first fusion model was a decision-based approach in which a linear regression model took the predictions of the CNN model and the random forest as input (*Figure 3A*, decision fusion without retraining). The random forest produced predictive probabilities for both classes, denoted as $p_{\mathrm{RF}0}$ and $p_{\mathrm{RF}1}$, which were fused with the class probabilities generated by CNN, $p_{\mathrm{CNN}0}$ and $p_{\mathrm{CNN}1}$. These probabilities were concatenated to form a four-dimensional vector:


$$p_{\mathrm{fused}}=[\;p_{\mathrm{RF}0},\;p_{\mathrm{RF}1},\;p_{\mathrm{CNN}0},\;p_{\mathrm{CNN}1}]$$

The linear regression model was fitted to the probability vector of the validation set, and the overall fusion model was evaluated on the test set.

Next, we employed the same pipeline but allowed retraining of the CNN model (*Figure 3B*, decision fusion with retraining). In this approach, the random forest model was again trained using the 417 handcrafted features extracted from ECG signals. This time, the probability vector was then fed into multi-layer perceptron (MLP) model for final classification. The MLP model is made up of a single linear layer with learnable weight matrix (W**)** and bias (*b*), and sigmoid activation function (*σ*), resembling the logistic regression fitted to the probability vector without retraining.


$$\hat{y}=\sigma (Wp_{\mathrm{fused}}+b)$$

Unlike the previous fusion approach, where a separate logistic regression model was fitted to the outputs of the models after training, the MLP in this setup allowed backpropagation through the CNN model. This setup ensured that the CNN could adjust its parameters based on the contribution of the random forest’s predictions, even though a random forest itself does not support backpropagation and therefore remains fixed. Consequently, the CNN learned to utilize both the ECG feature-based predictions made by the random forest and the temporal and spatial patterns learned from ECG median beats, leading to a more comprehensive decision-making process.

In the feature fusion process (*Figure 3C* and *D*), the CNN takes median beats computed from 12-lead ECG signals as input, following the standard pipeline. To extract meaningful feature embeddings, the fully connected layer of the prior CNN model was removed, and a 1 × 512 embedding vector was obtained from the Average Pooling layer. This approach preserved the temporal and spatial convolutions applied to the input signals and ensured that the extracted embeddings retained essential information.

For the first feature fusion approach (*Figure 3C*, embedding-feature fusion), the 1 × 512 CNN embeddings ($e_{\mathrm{CNN}}$) were concatenated with 417 temporally and spatially relevant features ($f_{417}$) extracted from ECG signals, which have demonstrated strong predictive capability in diagnosing ACS and OMI.^7,8^


$$f_{\mathrm{fused}}=\mathrm{concat}(e_{\mathrm{CNN}},\;f_{417})$$

This produced a fused feature representation of size 1 × 929, which was then used as input to a two-layer MLP model. The MLP architecture was fine-tuned to optimize performance on the validation set, ensuring effective integration of CNN-based and handcrafted features. This approach allowed the CNN model to generate meaningful embeddings incorporating the decision made from the feature fusion approach.

In the second feature fusion approach (*Figure 3D*, feature fusion with attention mechanism), we wanted to leverage the predictive power of the random forest classifier, which has demonstrated strong capability in learning and effectively utilizing handcrafted features extracted from ECG signals in our previous works. In this approach, we again implemented a random forest classifier, but this time, we extracted the predicted probabilities from each individual decision tree within the random forest model ($e_{\mathrm{RF}}$) and concatenated them with the CNN embeddings ($e_{\mathrm{CNN}}$), generated with ECG median beats as inputs. The decision to use predictions from individual trees rather than the aggregated random forest output was made to expand the feature space available to the MLP model. A study by Kong and Yu has shown that this approach can outperform traditional random forest predictions, as the MLP effectively learns a weighted combination of predictions from each tree.^28^ This provides greater flexibility in model decision-making compared to a traditional random forest, which relies solely on majority voting to determine the final classification. Again, this approach allows for the CNN to generate embeddings that incorporate the tree-based predictions from the random forest classifier through back propagation. We further use an attention-based approach to combine the CNN-derived embeddings with the random forest’s probability predictions here, allowing each feature representation to be weighted differently according to their relevance in the classification task. This method allows a parameter *α* to be dynamically learned that determines how much emphasis is placed on the random forest predictions and the CNN embeddings. Previous studies have shown the success of using attention-mechanism in fusion of information.^29,30^ Since the feature spaces of the embeddings and the random forest predictions have different dimensions, we projected both feature spaces into a common dimension, representing the fused features. These projections were performed through linear layers as follows:


$$e_{\mathrm{CNN}}=\mathrm{Linea}r_{\mathrm{CNN}}(e_{\mathrm{CNN}})$$

$$e_{\mathrm{RF}}=\mathrm{Linea}r_{\mathrm{RF}}(e_{\mathrm{RF}})$$

With the feature spaces having the same dimensions, it is simpler to concatenate them and pass them through an attention mechanism. The weight *α* is computed as follows:


$$\alpha =\sigma (\mathrm{Attention}(\mathrm{concat}(e_{\mathrm{CNN}},e_{\mathrm{RF}})))\;$$

Here, the concatenated feature spaces were passed into a small neural network with two layers, which outputs a scalar value for each sample, representing the attention weight. This scalar value was then processed by a sigmoid function to ensure that 0≤α≤1. The weight *α* was computed every time for each sample during the concatenation of the random forest and CNN projections. The final fused features $f_{\mathrm{fused}}$ were then represented by a weighted sum of the embeddings and random forest probabilities.


$$f_{\mathrm{fused}}=\alpha (e_{\mathrm{RF}})+(1-\alpha )(e_{\mathrm{CNN}})$$

Finally, the fused features are passed to a two-layer MLP model for classification, yielding the final prediction of the outcome.

The attention weight *α* in our fusion model can serve as an explainability mechanism and provide information on how the model integrates information from different feature sources to make its final prediction. Since *α* is dynamically computed for each input sample, it functions as an adaptive gating mechanism, determining the relative contribution of the CNN embeddings and random forest predictions. A higher *α* value indicates greater emphasis on random forest probabilities, whereas a lower *α* suggests that the CNN embeddings are more influential in the final prediction. This then provides a quantitative measure of how much ECG median beat representations and ECG-extracted features influence the decision-making process. By weighting these representations, the fusion model mirrors comprehensive real-world clinical reasoning, where physicians balance ECG waveform patterns with numerical spatiotemporal features to form a comprehensive assessment.^31^

Finally, to statistically compare the models’ test-set performances, we further generated 1000 bootstrap samples by resampling the test set with replacement and conducted statistical analysis using the Wilcoxon rank-sum test.

## Results

### Study cohort description

The data for this study were collected from three EMS systems across the USA. The cohort included 7397 patients and 10 393 ECGs, encompassing both initial and serial recordings. The cohort consists of 4825 ECGs from Pittsburgh EMS (Pittsburgh, PA), 5192 from Mecklenburg EMS, and 395 from Orange County EMS (North Carolina). Baseline characteristics of the study cohort are summarized in *Table 2*.

### Model performance

In this work, we proposed various hybrid models that fuse representations at both the feature and decision levels. *Table 3* presents the performance of our proposed hybrid models for the ACS and OMI outcomes, with metrics reported on both the validation and test sets. Corresponding boxplots for visual comparison are provided in the Supplementary section (*Figure 1*). The decision fusion with retraining approach achieved consistent performance on the test set for both outcomes, outperforming baseline models and other proposed hybrid approaches. Additional test-set performance metrics for the best-performing model are provided in the Supplementary section (*Table 2*). Overall, our proposed model achieved sensitivities of 0.648 and 0.774 for ACS and OMI, respectively, substantially surpassing a commercial device, which had sensitivities of 0.379 and 0.242.

**Table 3:** AUC and average precision (AP) scores of the proposed hybrid models and the baseline models, where the highlighted cells indicate the highest score achieved within each category

	Validation				Test			
Models	ACS		OMI		ACS		OMI	
	AUC	AP	AUC	AP	AUC	AP	AUC	AP
Baseline random forest	0.861	0.663	0.943	0.704	0.870	0.710	0.926	0.812
Baseline CNN	0.867	0.687	0.945	0.779	0.855	0.706	0.939	0.819
Decision fusion without retrain	**0**.**884**	0.711	**0**.**949**	**0**.**782**	0.878	0.732	0.939	0.838
Decision fusion with retrain	0.882	**0**.**713**	0.935	0.773	**0**.**878**	**0**.**749**	**0**.**960**	**0**.**848**
Embedding-feature fusion	0.872	0.695	0.916	0.748	0.861	0.732	0.943	0.816
Feature fusion with attention	0.849	0.655	0.939	0.724	0.861	0.704	0.925	0.804

*Table 4* provides a more comprehensive analysis of the bootstrapped samples, showing the mean and 95% confidence intervals of AUC and AP scores generated from the samples, as well as the pairwise comparisons between the decision fusion with retraining approach and all other models for both AUC and AP, and both outcomes. The decision fusion with retraining approach outperformed other models on the overall test set and showed a statistically significant difference compared to all others, except in the AUC metric when predicting ACS. This highlights the superior predictive power of such a modelling approach. Pairwise statistical comparisons of all models evaluated in this study, based on AUC scores on the test set, are presented in the Supplementary section (*Tables 3* and *4*).

**Table 4:** Mean and 95% confidence intervals of AUC and AP scores derived from bootstrapping the test set 1000 times with replacement

Models	ACS		OMI	
	AUC	AP	AUC	AP
Random forest	0.870 [0.832, 0.907]^a^	0.711 [0.641, 0.775]^a^	0.927 [0.877, 0.969]^a^	0.814 [0.718, 0.891]^a^
CNN	0.854 [0.808, 0.892] ^a^	0.706 [0.631, 0.774] ^a^	0.939 [0.901, 0.972]^a^	0.820 [0.730, 0.894]^a^
Decision fusion without retrain	0.878 [0.840, 0.913]	0.733 [0.662, 0.796]^a^	0.939 [0.894, 0.973]^a^	0.840 [0.752, 0.910]^a^
Decision fusion with retrain (reference for statistical comparison)	0.878 [0.837, 0.915]	0.749 [0.681, 0.810]	0.961 [0.934, 0.983]	0.850 [0.763, 0.918]
Embedding-feature fusion	0.861 [0.818, 0.901]^a^	0.733 [0.664, 0.798]^a^	0.944 [0.905, 0.976]^a^	0.818 [0.727, 0.895]^a^
Feature fusion with attention	0.861 [0.820, 0.900]^a^	0.705 [0.631, 0.770]^a^	0.926 [0.877, 0.967]^a^	0.807 [0.712, 0.886]^a^

^a^ *P* < 0.001, a significant difference between decision fusion with retraining against all other models on the test set using bootstrapped samples.

We also examined the attention weight (*α*) learned by the trained model in the fourth fusion approach, feature fusion with attention, to provide insights into how much the model emphasizes the embeddings from each individual model. On the test set, the model computed an average α of 0.958 for OMI and 0.812 for ACS, indicating that the model learned to put greater emphasis on the random forest embeddings.

## Discussion

In this study, we explored whether different fusion approaches, integrating ECG-based predictive models derived from our previous works, could further improve the AI-enhanced diagnostic prediction of ACS and OMI. Motivated by the observation that the base models performed differently across subsets of cases (*Figure 1*), we fused a CNN-based model with a feature-based random forest model using both decision-level and feature-level fusion strategies to optimize predictive accuracy. Among the evaluated fusion models, the decision fusion with a retraining approach achieved the best performance, outperforming the individual baseline classifiers as well as more complex fusion architectures. Feature fusion approaches outperformed individual models by some metrics, but their advantages were not consistent across all evaluations in comparison to the decision fusion approach.

Decision fusion hybrid models were the only models that consistently outperformed individual models, regardless of which individual model performed better. Even when individual models performed similarly across all test cases, they often made different errors. *Figure 1* shows that in many cases the predictions between the CNN and random forest models agreed (bottom left and top right quadrants) and were both correct. However, in a substantial minority of cases, the models make discordant predictions (top left and bottom right quadrants). These discordant quadrants contain both true positive and true negative cases, indicating neither model is completely accurate, and the two models make different types of errors. *Figure 2* further emphasizes this, illustrating different waveform patterns missed by each model.

Decision fusion leverages the complementarity of the two models by incorporating both sets of predictions, potentially reducing misclassifications and optimizing overall performance. Focusing on the two decision fusion approaches, one with retraining and one without, we find that decision fusion with retraining consistently outperformed both individual models and feature fusion. In ACS prediction, it achieved higher validation and test AP scores, while in OMI prediction, it improved both test AUC and AP. Even without retraining, decision fusion outperformed individual models by leveraging a weighted combination of their predictions.

Retrained decision fusion is particularly effective because it enables the model to adaptively learn how to combine the strengths of both approaches. When one model is stronger, retraining allows the fusion layer to increase its influence while still incorporating complementary signals from the other. For example, if the random forest outperforms the CNN, keeping the random forest fixed while allowing the CNN to adjust its outputs through backpropagation helps the fusion model align with the random forest’s decision boundaries. Conversely, when the CNN is stronger, the retraining process lets the model place greater emphasis on its more informative predictions while still benefiting from the random forest’s contributions.

Although feature fusion models were not the top performers overall, their statistical comparisons with the individual models still demonstrated a significant difference in certain metrics (*Tables 3* and *4* in the Supplementary section), and their results underscore the potential benefits of combining latent representations. In ACS prediction, the naïve feature fusion model achieved a validation AUC that exceeded both the CNN and the random forest, and a test AUC that surpassed the CNN only. In OMI prediction, while the naïve feature fusion model achieved a higher test AUC than either individual model, it did not outperform them across other metrics, making it unclear whether feature fusion provided a meaningful improvement in this case.

This pattern aligns with the relative strengths of the individual models. In ACS prediction, the random forest outperformed the CNN in both test AUC and AP score, highlighting the predictive power of handcrafted ECG features and the random forest model. Thus, fusing either these features or the predictions from random forest trees with the CNN embeddings improved performance compared to using the CNN alone. However, in OMI prediction, since the random forest did not outperform the CNN, combining their latent representations added limited value.

In summary, the substantial advantage of retrained decision fusion lies in its adaptability in dynamically prioritizing the stronger model while directly integrating useful signals from the weaker one through fusion at the decision level. This flexibility allows it to outperform other fusion approaches.

## Conclusion

In this study, we investigated the impact of fusion-based machine learning algorithms on the diagnostic performances of identifying two cardiovascular conditions, ACS and OMI, from the 12-lead ECG. We proposed four fusion approaches that built upon the random forest and CNN models described in our previous works, which demonstrated strong performance but tended to misclassify different subsets of cases. Model fusion was performed on either the feature level or the decision level and involved the baseline models as two parallel branches. Among all proposed approaches, the decision fusion with a retraining strategy achieved the best AUC and AP scores and significantly outperformed other models. This method offers a straightforward yet effective way to combine the predictive power of both models, allowing the CNN to directly incorporate the random forest model’s predictions in its own decision-making process. In summary, this work offers insights into different fusion approaches and underscores the potential of model fusion to enhance diagnostic precision in cardiovascular disease detection.

## Supplementary Material

ztag062_Supplementary_Data

## Data Availability

The ECG data used in this study will not be made publicly available due to the sensitive and confidential nature of health-related information.
