# Supplementary material for: Novel machine learning fusion architectures integrating electrocardiogram representations: applications to acute coronary event detection
Source: Eur Heart J Digit Health. 2026 May 8;7(5):ztag062. doi: 10.1093/ehjdh/ztag062 (PMC13188221; doi:10.1093/ehjdh/ztag062)
Supplement: ztag062_Supplementary_Data [file ztag062_supplementary_data.docx]

**Supplementary Materials**

**Table 1. Hyperparameters selection**

| **Hyperparameter** | **Search** | **ACS** | **OMI** |
| --- | --- | --- | --- |
| *CNN* | | | |
| Learning rate  (first epoch) | [1e-2, 1e-3, 1e-4] | 1e-3 | 1e-3 |
| Learning rate | [1e-4, 1e-5, 1e-6] | 1e-4 | 1e-4 |
| Batch size | [32, 64, 128, 256] | 32 | 32 |
| Weight decay | [1e-1, 1e-2, 1e-3] | 1e-3 | 1e-2 |
| *Random Forest* | | | |
| # estimators | [25, 50, 75, 100, 150, 200] | 50 | 25 |
| Criterion | [entropy, gini] | gini | entropy |
| Max features | [sqrt, log2, none] | sqrt | log2 |
| Min samples per split | [0.001, 0.005, 0.01] | 0.001 | 0.001 |
| Min samples per leaf | [0.001, 0.005, 0.01] | 0.001 | 0.001 |
| Min impurity decrease | [0.0, 0.001, 0.005] | 0 | 0.001 |
| Max samples | [0.25, 0.5, 0.75, 1] | 0.75 | 0.5 |
| Minimum cost complexity pruning alpha | [0, 0.001, 0.005] | 0.001 | 0.005 |
| Class weight | [balanced_subsample] | balanced_subsample | balanced_subsample |
| Bootstrap | [True] | True | True |
| OOB score | [True] | True | True |

**Figure 1. Boxplots comparing AUC and AP scores derived from bootstrapped evaluation across the proposed models for a) ACS and b) OMI outcomes.**

**a**


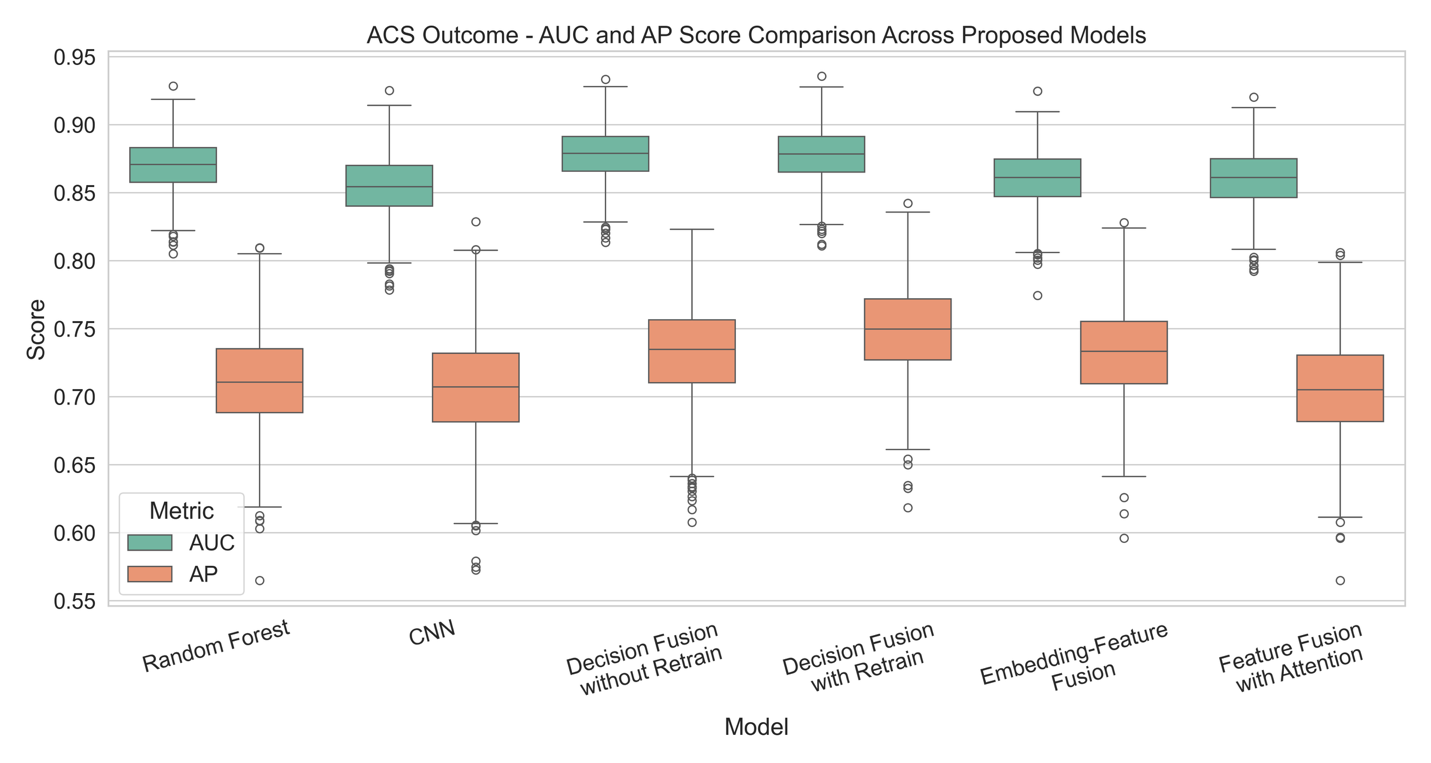


**b**

**
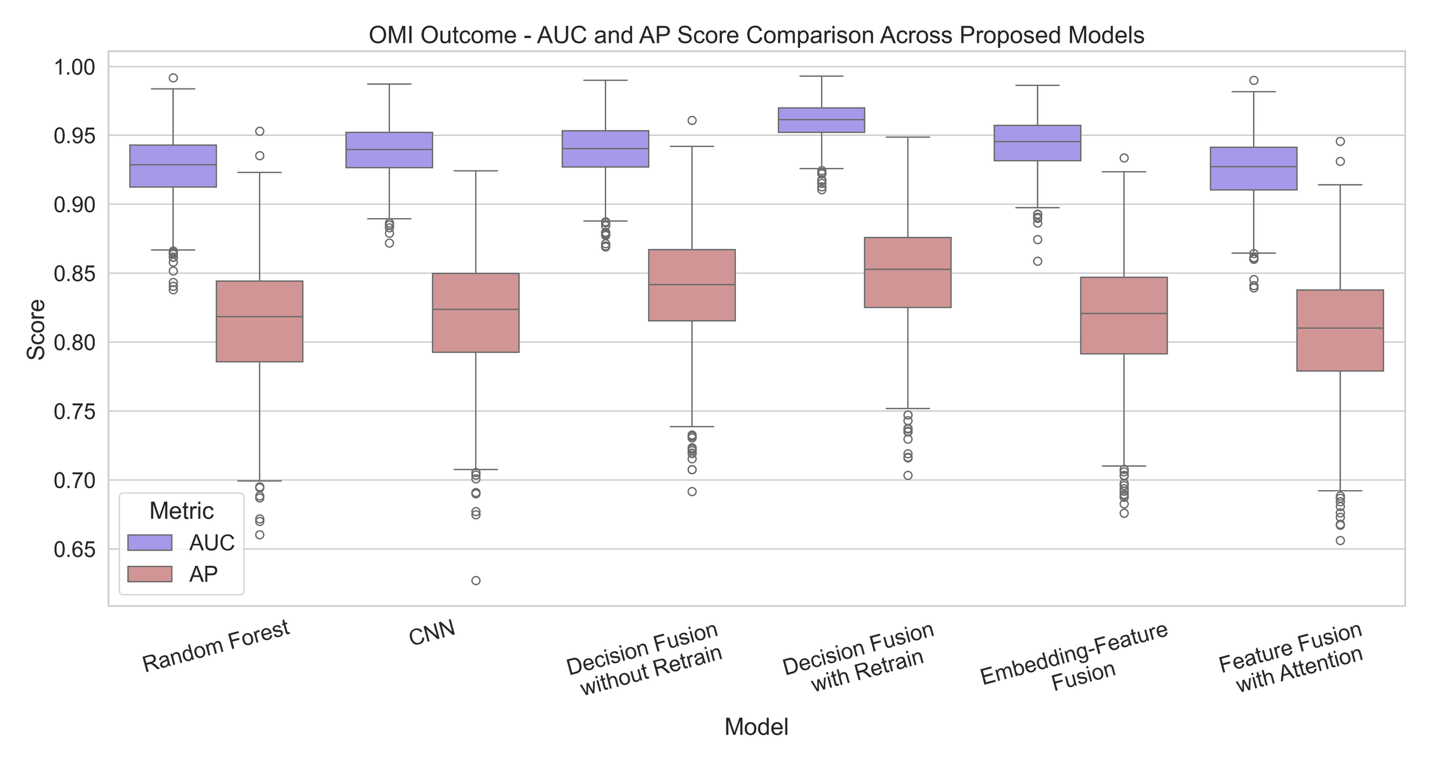
**

**Table 2. Additional test-set performance metrics of the model implemented with decision fusion with retrain approach, evaluated at threshold to maximize F1-score.**

|  | **Sensitivity** | **Specificity** | **Positive Predictive Value** | **Negative Predictive Value** | **F1-score** |
| --- | --- | --- | --- | --- | --- |
| **ACS** | 0.648 | 0.927 | 0.643 | 0.928 | 0.645 |
| **OMI** | 0.774 | 0.987 | 0.842 | 0.980 | 0.807 |

**Table 3. Statistical p-values computed from bootstrapped samples of the AUC scores on the test set using the Wilcoxon rank-sum test for the ACS outcome.**

|  | **Random Forest** | **CNN** | **Decision Fusion with Retrain** | **Decision Fusion without Retrain** | **Embedding-Feature Fusion** | **Feature Fusion with Attention** |
| --- | --- | --- | --- | --- | --- | --- |
| **Random Forest** | --- | p < 0.001 | p < 0.001 | p < 0.001 | p < 0.001 | p < 0.001 |
| **CNN** | --- | --- | p < 0.001 | p < 0.001 | p < 0.001 | p < 0.001 |
| **Decision Fusion with Retrain** | --- | --- | --- | p = 0.672 | p < 0.001 | p < 0.001 |
| **Decision Fusion without Retrain** | --- | --- | --- | --- | p < 0.001 | p < 0.001 |
| **Embedding-Feature Fusion** | --- | --- | --- | --- | --- | p = 0.980 |
| **Feature Fusion with Attention** | --- | --- | --- | --- | --- | --- |

**Table 4. Statistical p-values computed from bootstrapped samples of the AUC scores on the test set using the Wilcoxon rank-sum test for the OMI outcome.**

|  | **Random Forest** | **CNN** | **Decision Fusion with Retrain** | **Decision Fusion without Retrain** | **Embedding-Feature Fusion** | **Feature Fusion with Attention** |
| --- | --- | --- | --- | --- | --- | --- |
| **Random Forest** | --- | p < 0.001 | p < 0.001 | p < 0.001 | p < 0.001 | p = 0.213 |
| **CNN** | --- | --- | p < 0.001 | p = 0.570 | p < 0.001 | p < 0.001 |
| **Decision Fusion with Retrain** | --- | --- | --- | p < 0.001 | p < 0.001 | p < 0.001 |
| **Decision Fusion without Retrain** | --- | --- | --- | --- | p < 0.001 | p < 0.001 |
| **Embedding-Feature Fusion** | --- | --- | --- | --- | --- | p < 0.001 |
| **Feature Fusion with Attention** | --- | --- | --- | --- | --- | --- |
